# The impact of short-term confinement on human innate immunity

**DOI:** 10.1038/s41598-022-12380-5

**Published:** 2022-05-19

**Authors:** S. A. Ponomarev, A. A. Sadova, M. P. Rykova, K. D. Orlova, D. D. Vlasova, S. M. Shulgina, E. N. Antropova, O. V. Kutko, N. S. Germanov, V. S. Galina, V. A. Shmarov

**Affiliations:** 1 Laboratory of Immune System Physiology, SSC RF-IBMP RAS, Moscow, 123007 Russian Federation; 2 Pirigov Russian National Research Medical University (Pirogov Medical University), Moscow, 117997 Russian Federation

**Keywords:** Cytokines, Gene regulation in immune cells, Innate immune cells, Innate immunity, Lymphocytes, Immunology, Signal transduction

## Abstract

During space missions cosmonauts are exposed to a myriad of distinct stressors such as radiation, overloads, weightlessness, radiation, isolation in artificial environmental conditions, which causes changes in immune system. During space flights it is very difficult to determine the particular factor associated with the observed immunological responses. This makes ground-based experiments examining the effect of each space flight associated factor along of particular value. Determining mechanisms causing alterations in cosmonauts’ immunity can lead to potential targets for different countermeasures. In the current article we present the study of the early period of adaptation of human innate immunity of 6 healthy test-subjects, 4 males and 2 females aged 25 through 40, to isolation factors (hypodynamia, psychological stress, artificial environment). We measured multiple parameters characterizing innate immunity status in blood samples at chosen time points before, during and after the mission. In the experiment, highly enhanced cytokine responses were observed upon ex vivo antigen stimulations in comparison to baseline values. For cellular parameters we found multidirectional dynamics with a persistent prevalence of increasing TLRs^+^ monocytes as well as TLRs expression. Our study provides evidence that even a short-term confinement leads to immune changes in healthy humans that may trigger aberrant immune response.

## Introduction

Human immune system can be conditionally divided into two components constantly interacting with each other: innate and adaptive immunity. Innate immunity is basically activated upon the detection of pathogens, namely, viruses, bacteria, or pathogenic fungi to ensure the instant defense of the organism from these infection agents^1^. Innate immunity also triggers adaptive immune response^2, 3^. The key role in the detection of pathogens belongs to Pattern-recognition receptors (PRRs) capable of identifying conserved molecular structures of microorganisms and viruses, as well as some endogenous ligands, such as heat-shock proteins, fibrinogen, and several other high-molecular substances^4^. In the family of PRRs, there can be distinguished several groups of receptors with toll-like receptors (TLRs) being the largest and the most numerous. TLRs are widely expressed by immunocompetent cells, including monocytes, macrophages, dendritic cells, mast cells, eosinophils, basophils, neutrophils, T- and B-lymphocytes^5, 6^. Alterations in the functioning of TLRs in innate immunity cells can provoke the development of severe pathologies such as autoimmune diseases and immunodeficiency^6, 7^.

Today, it is common knowledge that extreme factors of space flights may lead to a number of functional changes in the state of various physiological systems of the human body, including the immune system^8^. Numerous experiments held during and upon the completion of space missions demonstrated alterations in the adaptive component of the human immunity manifested in the quantitative changes of T- and B-lymphocytes, the decrease in the T-cells functional activity, alterations in the blood serum cytokine profile, the shift in the Th1-Th2 cytokine balance to Th2 mainly which is in charge with humoral response development, and in the reactivation of latent intracellular viral infections^9–11^. In addition, significant changes have been revealed in the IgM repertoire, stable up to 30 days after mission completion^12^. Space flight conditions significantly affect innate immunity: the increase in the number of granulocytes with the simultaneous decrease in the monocytes relative quantity have been demonstrated. The decline in the IL-6 synthesis and CD62L and HLA-DR antigens expression by monocytes have been also reported along with a noticeably reduced secretion of IL-10, IL-6, and TNF-α by activated monocytes in vitro^13^.

Changes in the immune system similar to those revealed during space flights are also observed in ground-based model experiments (missions) with confinement in a hermetically sealed chamber with a closed environment. All experiments could be conditionally divided into two groups: long (over 21 days) and short-term (up to 21 days) missions.

### Long-term missions (“Mars-500” project)

#### 105-day experiment

Strewe C. et al. in the 105-day confinement experiment revealed the increase in reactive oxygen species production by polymorphonuclear leukocytes after TNF-α and fMLP stimulation on days 34 and 66 of the mission with the simultaneous depression of the phagocytic activity in peripheral blood of 6 male test-subjects^14^.

#### 520-day experiment

In the 520-day isolation, there were demonstrated an increased synthesis of the lymphocytes early activation marker CD69, a decreased functional potential of the NK-lymphocytes along with a decline in the absolute and relative content of the TLR2^+^, TLR4^+^, TLR6^+^-monocytes during practically the entire mission starting from day 120. At the same time, the relative number of granulocytes expressing the above-mentioned receptors did not change so dramatically: a decrease in the absolute and relative content of TLR6^+^-cells was demonstrated on days 120 and 360 of the experiment^15^. The relative number of granulocytes significantly decreased on days 360 and 410 of the mission, although their absolute number remained unchanged. Absolute number of CD19^+^ cells were increased on the same time points as well as CD3^+^ lymphocytes. Absolute count of CD3^+^CD8^+^ was increased on the 360 day of the mission while CD3^+^CD4^+^ and NK-cells remained unaltered during the mission. The production of TNF-α and IFN-γ by peripheral blood mononuclear cells after the stimulation by Epstein-Barr-Virus -lysate was increased on the 360th, 410th, and 510th days of the mission. The plasma concentration of the cytokines revealed no significant difference compared to the baseline period^16^.

In both of the experiments the crew consisted of 6 male volunteers.

### Short-term missions

#### 10-day mission

In this experiment, there participated 5 male test-subjects. An increased percentage of monocytes was observed on the 7th day of the mission in comparison to baseline values. The relative number of lymphocytes was decreased on the 7th and 10th days of confinement and remained decreased during the whole post-isolation period (5 and 7 days after the end of the mission). Granulocytes on the contrary were elevated starting from the 7th day of the mission till the 7th day of the recovery period. NK cells and CD69+ NK cells were reported to be increased starting from the 7th day of the mission remaining significantly increased even on the 7th day of recovery period^17^.

#### 17-day mission

During the experiment, within the framework of the SIRIUS international project, a significant decrease in monocytes expressing intracellular (TLR3, TLR8, TLR9) and surface (TLR4, TLR5, TLR6) TLRs was demonstrated on day 7 after the completion of the exposure, while no considerable changes were observed during the experiment. By the 7th day of the mission an increase in the number of CD14-CD16-CD123+CD85k+ plasmacytoid DCs was observed^18^.

On the whole, except for some works, at present there are no comprehensive studies describing TLR system functioning throughout the early adaptation period of confinement. In this regard, the current study is aimed at the complex investigation of the TLR system of monocytes during a 14-day confinement.

## Materials and methods

### Subjects and mission

The study was reviewed and approved by the Biomedicine Ethics Committee of the RF SRC-Institute of Biomedical Problems, Russian Academy of Sciences/Physiology Section of the Russian Bioethics Committee Russian Federation National Commission for UNESCO (protocol №573 from April 1, 2021). All participants provided their written informed consent to participate in this study. All methods were performed in accordance with the relevant guidelines and regulations.

**Figure 1:**
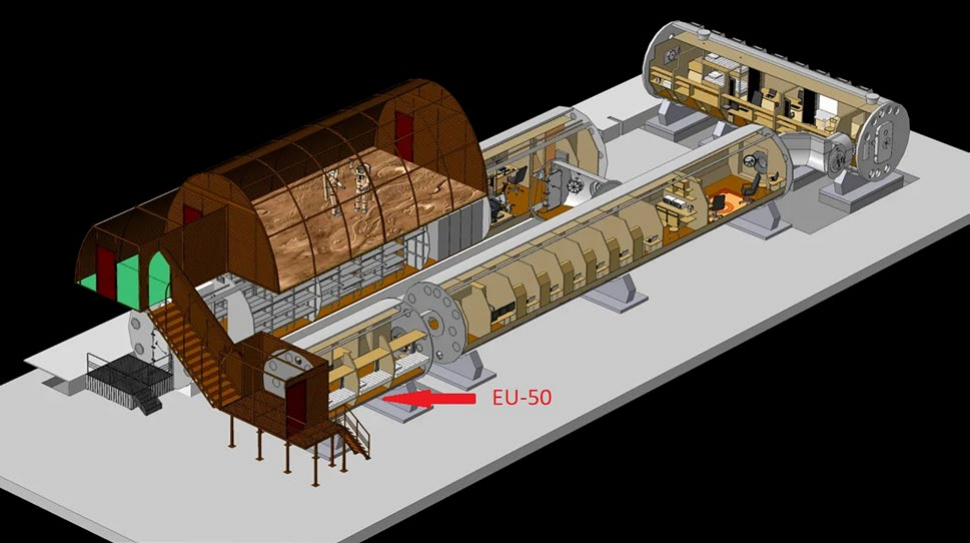
The scheme of the ground-based analog facility (NEK) at the Institute of Biomedical Problems of the Russian Academy of Sciences in Moscow. EU—experimental unit. [Hobihojin H. The transection of the NEK facility. Scheme. IBMP RAS. *Mars500 project*. [online]: http://mars500.imbp.ru/nek.html [Accessed 22 Nov 2021].

Fasting blood samples from each timepoint were collected in the morning from six almost healthy test-volunteers (4 males and 2 females) aged 25 to 40. Blood was sampled before the confinement (−7 days and −2 days, baselines 1 and 2, correspondingly), during the mission (days 3, 7, 14 of the confinement), and during the recovery period (7 days after confinement completion). The crew was confined in a hermetically sealed chamber with the isolated artificial environment with the approximate area of 50m^3^ in the ground-based analog facility called "Nazemnyy Eksperimental’nyy Kompleks" (Ground-based Experimental Facility), or NEK, at the Institute of Biomedical Problems of the Russian Academy of Sciences in Moscow (Fig. 1). During isolation different environmental parameters, including temperature (22–24 ^0^C), relative humidity (40–50), air pressure was 0,3 kPa higher than standard atmosphere, normal atmosphere gas composition, were kept at constant range. Blood was sampled from the cubital vein according to the standard technique under aseptic conditions into vacuum tubes from Greiner Bio-One (Austria) with a standard content of anticoagulants (K_3_-EDTA and sodium citrate 3.8%) and without them to collect blood serum.

### Examined parameters

The immunological studies included the investigation of the following parameters of the immune system:

- The content of leukocytes, lymphocytes, monocytes, and granulocytes in the peripheral blood;
- The content of monocytes expressing surface TLRs (TLR1, TLR2, TLR4, TLR5, TLR6) and intracellular TLRs (TLR3, TLR8, TLR9) in the peripheral blood;
- The level of TLRs expression by monocytes;
- The level of TLRs expression by monocytes in vitro after stimulation;
- The levels of GM-CSF, IFN-α2, IFN-γ, IL-10, IL-12P40, IL-12P70, IL-1β, IL-6, IL-8, TNF-α, MCP1, GRO cytokines in the blood serum;
- The synthesis of cytokines (GM-CSF, IFN-α2, IFN-γ, IL-10, IL-12P40, IL-12P70, IL-1β, IL-6, IL-8, TNF-α, MCP1, GRO) in monocytes cultured for 24 h and stimulated with corresponding TLRs and NLRs ligands described in details below.
- Gene expression: TLR1, TLR2, TLR3, TLR4, TLR5, TLR6, TLR8, TLR9, HSPA1A, TICAM2, HSPD1, FOS, IL1B, IL12A, CSF2, IL6, IL8, IL10, LTA, IRF1.

TLRs and NLRs ligands were used from TLR1-9 Agonist kit and NOD1/2 Agonist Kit (Invivogen, USA) according to the manufacturer’s instruction.

The content of leukocytes, as well as the absolute and relative number of lymphocytes, monocytes, and granulocytes in the peripheral blood were determined using automatic hematology analyzer Celltac-α MEК 6318 K (Japan).

### Flow cytometry analysis

The analysis of the receptor structures of immunocompetent cells was carried out by a multiparametric method of immunofluorescence analysis using a panel of monoclonal antibodies, which included monoclonal antibodies to TLR1 (CD281(TLR1)-PE, eBioscience, USA), TLR2 (CD282(TLR2)-FITC, eBioscience, USA), TLR4 (TLR4-FITC, Hycult Biotech, Netherlands), TLR5 (TLR5-PE, Invitrogen, USA), TLR6 (TLR6-PE, Hycult Biotech, Netherlands). The samples for the intracellular TLRs assessment were treated with IntraPrep Permeabilization Reagent (Beckman Coulter, USA) in accordance with the manufacturer’s instructions prior to the incubation with the corresponding monoclonal antibodies (MCATs). Then, to control and test the samples of intracellular TLRs, the following MCATs were added: TLR3 (CD283(TLR3)-PE, eBioscience, USA), TLR8 (TLR8-PE, Invitrogen, USA), TLR9 (CD289(TLR9)-PE, eBioscience, USA), cell suspensions were resuspended and incubated for 30 min at 4 °C, then washed with the ice-cold buffer, resuspended in 350 µl of the buffer and analyzed using flow cytometry.

**Figure 2:**
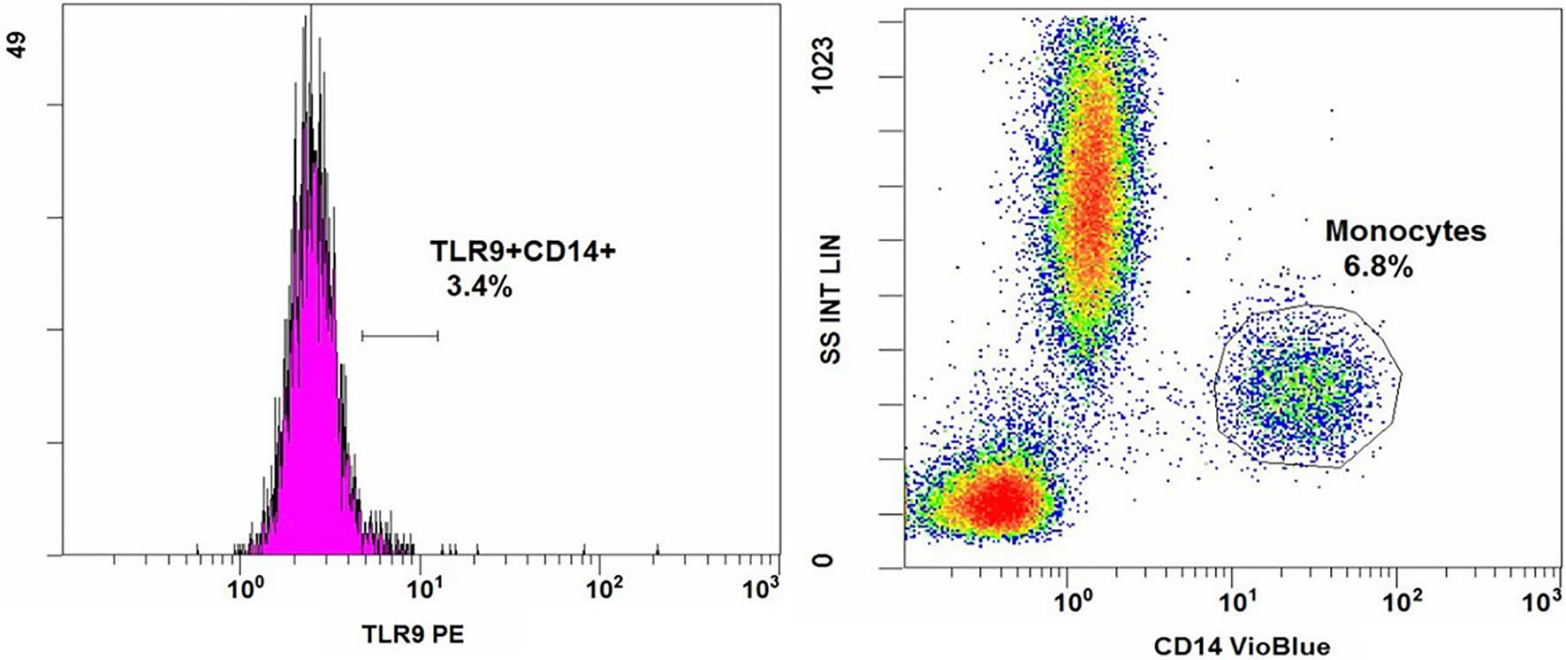
Flow cytometry analysis.

To determine the expression of the receptors localizing on the membranes of immunocompetent cells, 10 µl of monoclonal antibodies were added to 50 µl of blood with K_3_-EDTA and incubated for 20 min at room temperature. Then, 500 µl of lysis buffer BD FACS Lysing (Becton Dickinson, USA) were added to each of the tubes and incubated for 10 min at room temperature to lyse erythrocytes. After two subsequent washes of cell suspensions from unbound antibodies and erythrocyte debris with CellWash (Becton Dickinson, USA) the cells were centrifuged at 300 g for 5 min at room temperature. The stained cells were counted using BC Navios™ Flow Cytometer (Beckman Coulter, USA) (Fig. 2).

### MNC separation

Mononuclear cells (MNCs) were isolated from heparinized venous blood by centrifugation in the density gradient using Ficoll (ρ = 1077). The monocultures of CD14^+^-cells were obtained from the suspensions of MNCs using MS Columns magnetic separation (Miltenyi Biotec, Germany), human FcR Blocking Reagent (Miltenyi Biotec, Germany), and human CD14 MicroBeads (Miltenyi Biotec, Germany) according to manufacturer’s instructions.

For the investigation of the impact of TLR ligands to the functional activity of monocytes CD14^+^-cells were supplied with RPMI-1640 full medium (Gibco, USA), containing 2 mM L-gln (Gibco, USA), 1% penicillin/streptomycin (Gibco, USA), 5% inactivated FBS (HyClone, USA), diluted to the concentration of 5*10^5^ cell/ml, and planted 200 µl per well to 96-well culture plates (Eppendorf, Germany). For each of the TLRs investigated there were control samples in which the corresponding ligand was not added. The ligands for surface and intracellular TLRs were taken from commercial Human TLR1-9 Agonist kit (Invitrogen, USA).

For the investigation of the impact of NLR to the functional activity of CD14^+^-cells were supplied with the full medium, diluted to the concentration of 1*10^6^ cell/ml, and planted 500 µl per well to 24-well culture plates (SPL Lifesciences, Korea). The ligands for NLRs were taken from the commercially available Human and mouse NOD1/2 Agonist Kit (Invitrogen, USA. No ligands were added to the control samples.

The CD14^+^-monocytes were cultured for 24 h at 37 °C, 5% CO_2_, culture supernatants were collected and stored at –80 °C until analysis.

The cultured cells stimulated with TLRs ligands were used to determine TLRs expression levels by flow cytometry analysis. The cell suspensions from the 96-well plate were washed with a buffer, containing DPBS (Gibco, USA), 2 mM EDTA (Sigma-Aldrich, USA) and 5% BSA (Miltenyi Biotec, Germany). The cell pellets were resuspended in 60 µl of the buffer with the addition of human FcR Blocking Reagent (Miltenyi Biotec, Germany) and incubated for 10 min at 4 °C to reduce non-specific binding of MCATs. To all samples on ice, MCATs same as in whole blood analysis were used. The cell suspensions were resuspended and incubated for 30 min at 4 °C, then washed with the cooled buffer. To collect cells from the culture plates 200 µl of the buffer were added and cells were pipetted on ice 80–100 times, then transferred to 12 × 75 mm tubes, the samples volumes were adjusted to 350 µl with the buffer, and the samples were analyzed using flow cytometry.

### Cytokine analysis

Cytokine production by the cultured cells was estimated using multiplex analysis and the commercially available panel for simultaneous cytokine and chemokine detection MILLIPLEX MAP Human Cytokine/Chemokine Magnetic Bead Panel—Premixed 41 Plex—Immunology Multiplex Assay (Merck Millipore, USA) according to manufacturer’s instructions.

### Stress hormones measurment

Blood serum cortisol was determined using commercially available DBC kit (Diagnostics Biochem Canada, UK). Adrenaline and noradrenaline levels in the plasma were measured with CatCombi ELISA kit (IBL international, Germany).

### Gene expression

ExtractRNA reagent (Evrogen, Russia) was used for phenol–chloroform extraction of total RNA from CD14^+^ monocytes according to the manufacturer’s instruction. RNA was precipitated in isopropanol with the addition of Satellite Red (Evrogen, Russia), dissolved in RNase-free water and DNase treated with DNase I (Thermo Fisher Scientific, USA). RNA quantity was assessed on NanoDrop 2000C (Thermo Scientific, USA) and the quality control of the isolated RNA was performed using gel electrophoresis. Reverse transcription was performed in the reaction volume of 10 µL using MMLV RT kit (Evrogen, Moscow, Russia), aliquots of total RNA were reverse transcribed using Oligo-dT primer and 100 units of MMLV RT at 42 °C for 60 min. The comparative C_T_ experiment was performed on AB StepOnePlus Real-Time PCR system (Thermo Fisher Scientific, Carlsbad, CA, USA) using SYBR Green reagent qPCRmix-HS SYBR + HighROX (Evrogen, Moscow, Russia) with the reaction set up according to the manufacturer’s instructions. The reactions were performed in triplicates and included negative controls in which RNA was added as template. The run protocol consisted of 40 cycles of 95 °C for 15 s, 60 °C for 15 s, and 72 °C for 30 s, with the fluorescence intensity measurement performed after the elongation stage. The specificity of PCR products was confirmed using melting curve analysis. RNA from -7 day monocytes was used as reference sample, and the expression of target genes was normalized to that of TBP. The analysis of fold difference in the expression of target genes was performed with StepOne Software v2.3 (Thermo Fisher Scientific, USA), MS Excel 2010.

### Statistical analysis

The results were analyzed using Statistica v.10.0 for Microsoft Windows software and assessed using the Wilcoxon signed-rank test. Differences between time points were considered significant if p < 0.05 and are indicated with an asterisk (*) on each data figure and table. The results are presented as Me, q25, q75.

## Results

In the course of the experimental study, it was shown that 14-day isolation in a pressurized chamber with an artificial environment had a significant effect on the cellular and humoral components of the immune system of the human body. These observations are based on the measurements of the absolute and relative content of immune cells in peripheral blood, as well as the assessment of the expression of the specific markers of innate immunity. Here, the relative content of cells means the percentage of cellular factors with a given phenotype contained in a particular cell pool, while the absolute number is the total number of cells with a certain phenotype per unit volume. The absolute and relative cell counts are important characteristics of the cellular immunity.

## Flow cytometry analysis

### Cell subsets in the peripheral blood

The analysis of the monocytes in peripheral blood revealed a significant increase in the absolute and relative content of CD14^+^-cells on the 3rd, 7th and 14th days of confinement preserving the increased values compared to the baseline data on −7 and −2 days (Supplementary Table S1). Meanwhile, the number of TLR1-expressing monocytes had multidirectional dynamics throughout the experiment, both in absolute and relative values. Undulatory changes in the expression of TLR1 were also observed. TLR2 expression significantly increased on the 3rd day of the mission compared to both baseline values. The absolute content of TLR2^+^-cells compared to baseline significantly increased starting from the 3rd day of the project. On the 3rd, 7th and 14th days there was an increase in the TLR4^+^-cells absolute count. Their relative number had already significantly increased by day −2 and preserved elevated by the 3rd day of the confinement. The percentage of CD14^+^TLR6^+^-cells significantly increased on the 3rd day compared to baselines 1 and 2, and their absolute content increased on the 7th and 14th days compared to the baselines.

The content of monocytes expressing intracellular TLRs also varied during the mission. Although the TLR3^+^-monocytes quantity did not change significantly during the confinement, the expression of TLR fluctuated spasmodically. The relative content of TLR8^+^-monocytes had a pronounced tendency to decrease except for the 14th day of the confinement and the recovery period, when a significant increase in their content compared to the baselines was noted. The same dynamics were observed for their absolute values. The expression level of TLR8 also decreased and increased significantly in the corresponding timepoints. For TLR9^+^-monocytes, we registered a significant increase in the expression of TLR9 on the 14th day of the confinement, and its levels remained elevated by the + 7 day of the experiment. The same was observed for the relative content of TLR9^+^-monocytes in the peripheral blood compared to the baselines.

Due to the bioethical restrictions concerning blood sampling, the quantity of biological samples collected did not allow to extract enough cells to estimate the ability of monocytes to produce cytokines on the 3rd day of the mission and two days before the confinement (baseline 2).

### Cell cultures

The stimulation of the cultured monocytes with TLRs ligands altered the expression of both surface and intracellular TLRs (Supplementary Table S2). First, we assessed the significance of these changes compared to control samples, and then these data were compared to the baseline values. The data which differed from both control and baseline were considered significant, and the same approach was applied to the analysis of induced cytokines synthesis.

As a result, this experiment demonstrated a significant increase in the percentage of TLR1-expressing monocytes by the 7th day of the confinement completion, while the content of TLR2 and TLR6, on the contrary, decreased significantly. There was a significant decline in the TLR2 expression level by the recovery period compared to baseline 1. The expression of TLR4 significantly increased by the 14th day of the confinement. Among the intracellular TLRs, only the expression of TLR8 changed at the 7th day of the experiment, while other TLRs did not demonstrate any alterations in the expression.

### Gene expression

**Figure 3:**
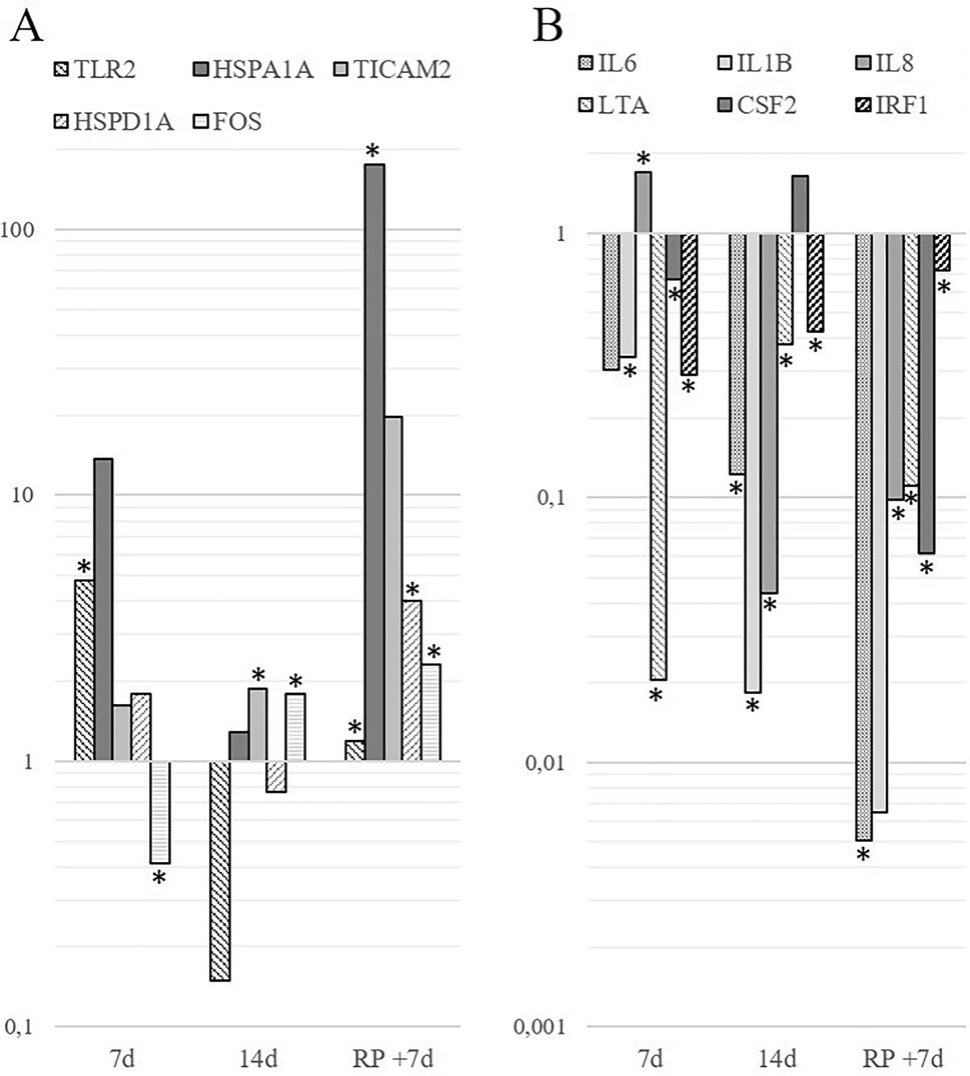
The relative expression (log10) of the genes of interest in the monocytes extracted from the peripheral blood at the indicated time points normalized to the expression of TBP as reference gene and baseline 1 (−7d) as reference sample (ΔΔC_T_ method). (**A**) The relative expression of genes encoding TLR2, TLR adapter molecules (TLR2, HSPA1A, TICAM2, HSPD1, FOS genes). (**B**) The relative expression of IL6, IL1B, IL8, LTA, CSF2, and IRF1genes, which encode the participants of TLRs’ signaling cascades. Asterisk (*) indicates *p*-value ≤ 0,05 in Wilcoxon test.

Molecular biology studies focused on the gene expression analysis managed to show the statistically significant changes of expression for the genes encoding TLR2, TLR adapter molecules (TLR2, HSPA1A, TICAM2, HSPD1 genes) (Fig. 3A), and the proteins expressed in the pathways induced upon TLRs signaling activation (FOS, IL1B, CSF-2, IL6, IL8, LTA, IRF1 genes) (Fig. 3B) compared to baseline 1.

TLR2 gene expression dramatically increased fivefold by day 7 of confinement compared to the baseline value, yet it was normalized in the recovery period of the experiment. On the contrary, the expression of HSPA1A gene was increased 175-fold by the completion of the experiment, while during the confinement its changes were rather uneven and statistically non-significant. The quantity of TICAM2 transcripts doubled by the 14th day of the confinement and increased almost 20-fold by the day + 7. The levels of HSPD1A gene expression demonstrated a similar pattern: they were increased on the days 7 and + 7 of the experiment. The changes in the expression of FOS gene were statistically significant during the whole observation period: by the 7th day of the confinement it had decreased twofold but gradually increased from this point reaching its peak by the day + 7.

A statistically significant decline in the expression of IL6 gene was observed during the whole experiment with the maximum (200-fold) decrease by day + 7. Similarly, the expression of IL1B was gradually decreasing during the experiment reaching its minimum by the day + 7, when it was 160-times lower than the reference value. The IL8 gene expression dramatically decreased 25-fold compared to baseline period after a two-fold increase by the 7th day of the confinement. The gene expression level of LTA was downregulated during the whole experimental period, in which it demonstrated a drastic decrease by the 7th day of the confinement. For IRF1 we noted the following fluctuations of expression: from fivefold decrease on the 7th day of the confinement to the smooth rise and normalization to baseline values by the recovery period. Spasmodic changes in the expression of CSF2 gene were observed: by the 7th day of the confinement the level of its expression was slightly declined with a subsequent dramatic two-fold increase by the 14th day, and by the day + 7 in the recovery period the expression of this gene was significantly downregulated.

The expression of other genes was measured, yet, the data appeared to be non-significant after the statistical analysis.

### Serum cytokines

**Figure 4:**
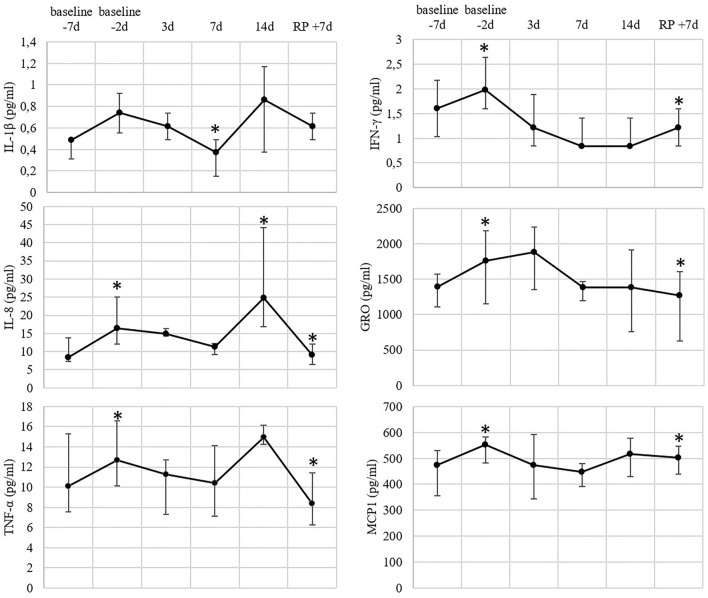
Blood serum cytokine levels in the test-volunteers at the indicated time points. Markers represent medians, whiskers represent quartiles q75 and q25; asterisk (*) indicates *p*-value ≤ 0.05 in Wilcoxon test.

Concerning the serum cytokine levels, the significant fluctuations for those of IL-1β, IL-8, TNF-α, IFN-γ, MCP1 were demonstrated (Fig. 4). The concentration of IL-1β decreased on the 7th day of the confinement and IL-8 was differentially synthesized during the baseline period, and on the 14th day of the confinement compared to baseline 1. The concentration of TNF-α was significantly elevated from baseline 1 to baseline 2, and decreased in the recovery period. The level of IFN-γ was increased by baseline 2 and smoothly declined upon the completion of the experiment by day + 7. The same tendency was observed for MCP1: its concentration was risen by baseline 2 and gradually decreased during the confinement.

### Cytokines production in cell cultures

During the experiment both basal cytokine expression by the peripheral blood monocytes and the expression of cytokines induced by TLRs ligands underwent significant changes (Supplementary Tables S3, S4). Considering cytokines spontaneous synthesis, GM-CSF level was higher on the 7th day of the confinement compared to baseline 1, while IL-1β concentrations significantly decreased by the 3rd day with the minimum on the 14th day of the confinement. IL-6 and TNF-α levels were also significantly declined by the same timepoint. On the contrary, a dramatical increase in the IL-8 level was noted on the 7th day of the mission. All the changes observed were significant compared to baseline 1 values.

**Figure 5:**
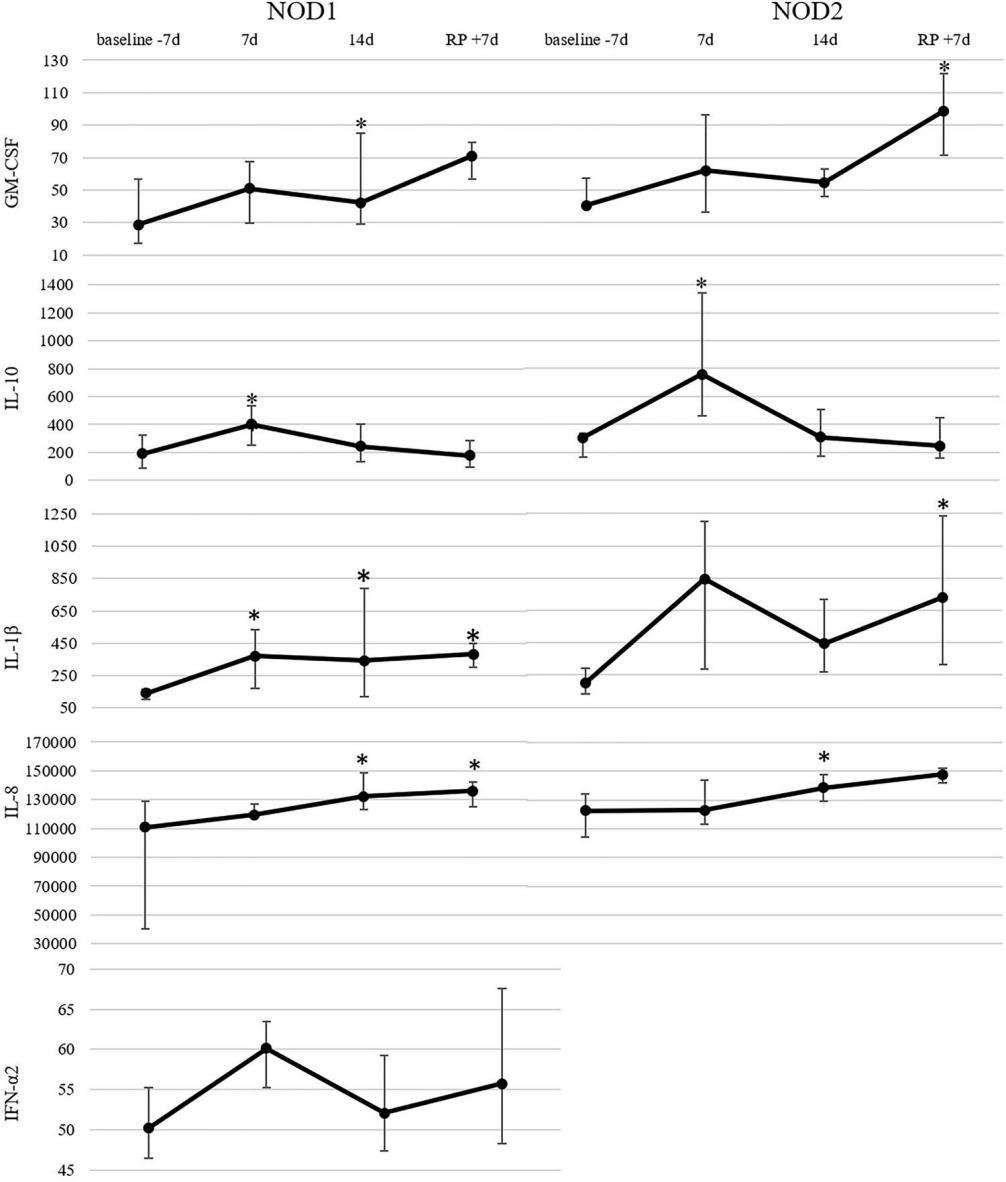
Induced cytokine synthesis in cultured monocytes stimulated with the corresponding NOD1 or NOD2 ligands. All values are presented in pg/ml. Markers represent medians, whiskers represent quartiles q75 and q25; asterisk (*) indicates p-value ≤ 0,05 in Wilcoxon test.

As for induced cytokine synthesis, a general tendency to the increased expression of all cytokines investigated was noted during the whole experimental exposure with significant difference compared to baseline values. Interestingly, the number of significant changes in cytokines synthesis upon NLRs stimulation was noticeably lower compared to those upon the stimulation of TLRs (Fig. 5).

### Stress hormones

**Figure 6:**
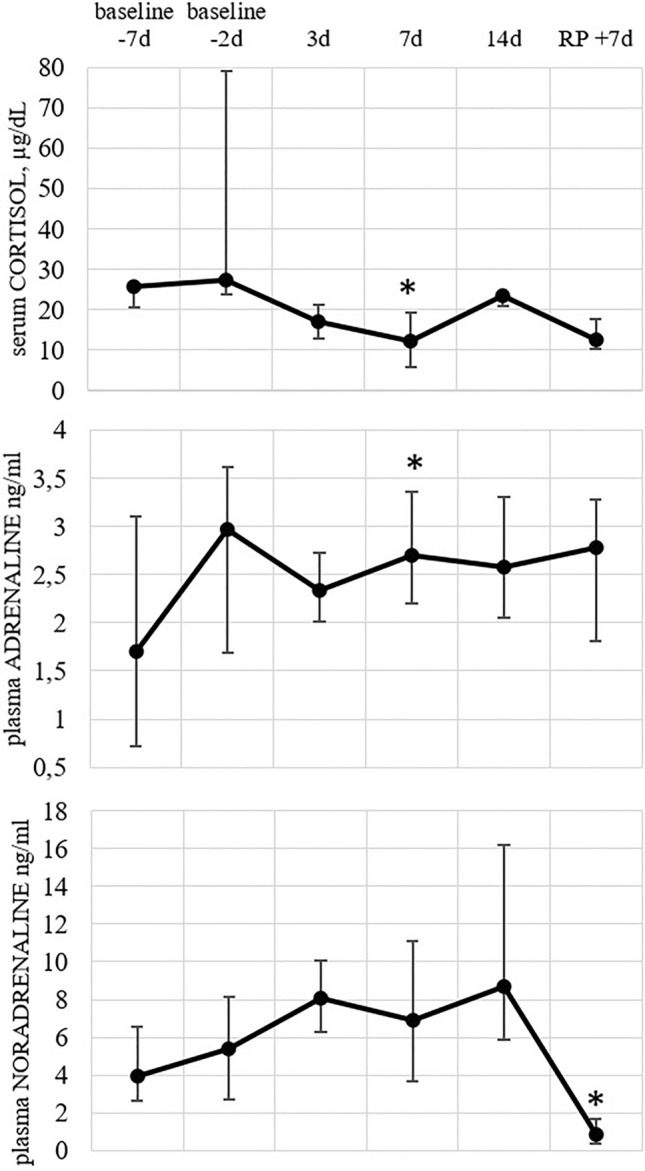
Blood hormone concentrations at the indicated timepoints. Markers represent medians, whiskers represent quartiles q75 and q25; asterisk (*) indicates *p*-value ≤ 0.05 in Wilcoxon test.

Adrenaline and cortisol concentrations in the blood were elevated by the 7th day of the mission, while there were no significant changes in the concentration of noradrenaline during the whole experiment (Fig. 6). Though, its concentration lowered in the recovery period, day 7 after the completion of the confinement.

## Discussion

Overall, the experimental findings demonstrate that short-term confinement leads to a number of changes in the human innate immunity manifested in increased cytokines level in serum, elevated spontaneous and induced cytokines production by monocytes, changes in TLRs signaling pathways and alteration of TLRs^+^ monocytes content in the peripheral blood as well as monocytes TLRs expression in blood and cellular cultures.

**Figure 7:**
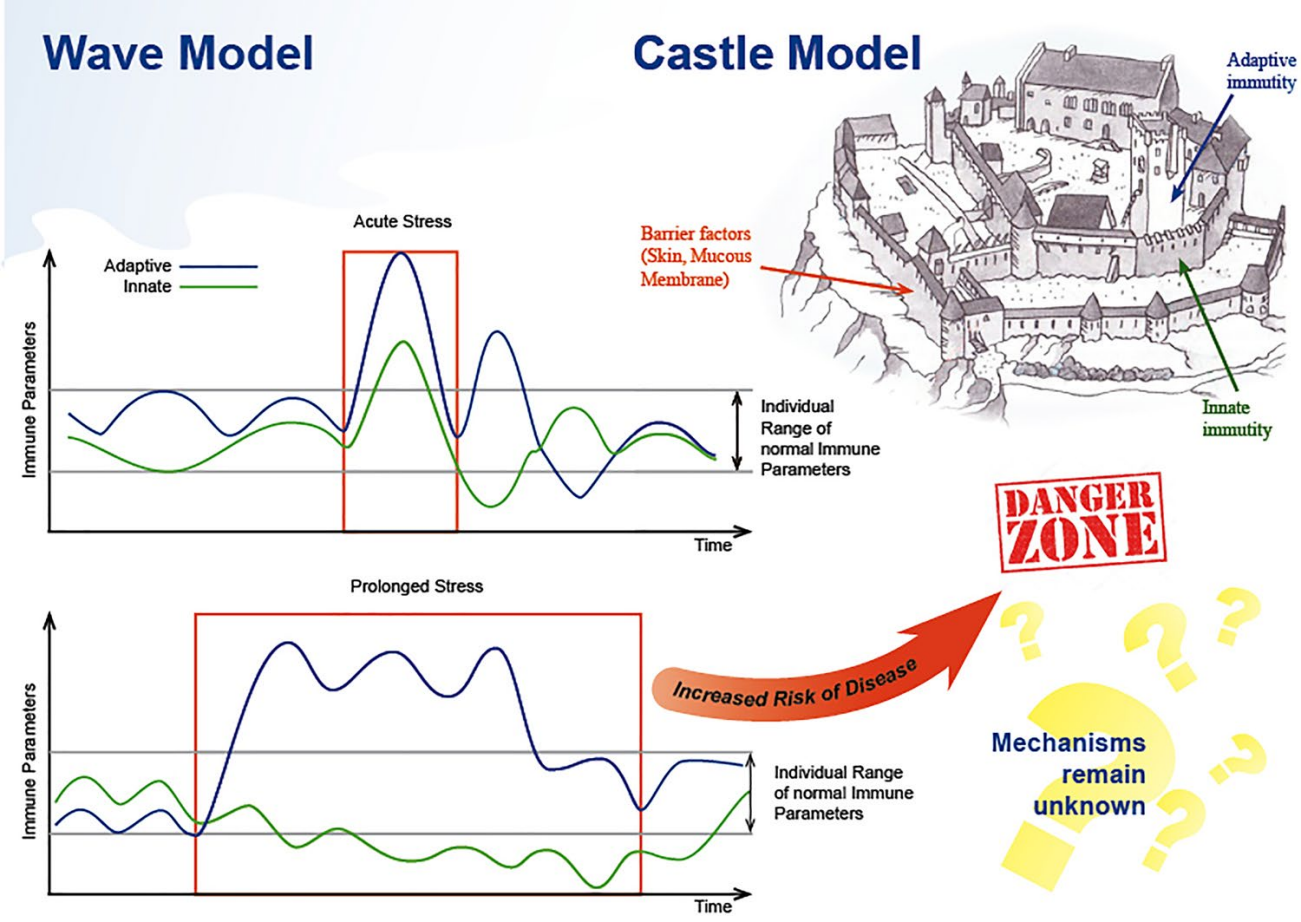
The two models of immune system functioning.

The influence of the factors associated with space flight on the human immune system is one of the courses of the global issue over the investigation of environmental conditions impact on human immunity. In these terms, the human immune system is affected specifically and non-specifically. The former effects include the interactions of immunity with different kinds of bacteria and viruses, or the endogenous structures, e.g., transformed tissues and cells, resulting from neoplastic growth. In these conditions, the development of the immune response leads to the elimination of the antigenic structures. In case of non-specific effects, one can observe the activation of different components of the immune system inducing the alterations in its functioning. The immune system functioning can be simplistically represented as the two models: the castle and the wave ones (Fig. 7). The former consists of a number of physiological barriers on the way of an infectious or a non-infectious pathogen, starting from barrier tissues and protective humoral factors, and ending with innate and adaptive immunity, in which the adaptive immune system is an ultimate bulwark in case of the rest of the resistance systems fails. The wave model illustrates the natural fluctuations (cycles of rise and fall) of the immunity parameters in the course of time and establishes the individual range of their shifts. The immune system is in balance with internal and external environment, which determines the natural fluctuations of its parameters, however, under the prolonged severe action of stress factors of various nature they exceed the normal individual range and cause the increase in the risk of the development of immunodeficiencies^19, 20^ or autoimmune diseases^21, 22^. The mechanisms by which the environmental factors affect human immune system have been poorly studied so far. To date, there exist several theories which explain how environmental factors influence human body and, in particular, the immunity. According to the first one, the action of the stress factors is realized through the neuroendocrine system^23, 24^. The second one adduces strong evidences of direct influence of the environment on the cells. In this case, a vivid example is either a real or a simulated alteration of gravity^25, 26^, as well as hyperbaric influence^27, 28^. Different gravity levels affect the immunity cells via the impairment of cytoskeleton structure, the involvement of mechanosensory ion channels^29^, and also via the enzymatic activity alteration of the participants of intracellular signaling cascades^30^.

We speculate that in the performed experiment the changes in TLR system were basically caused by psychological stress. Below the alterations identified are considered by groups.

### Cell subsets in the peripheral blood and in-vitro stimulation

Our study revealed the heterogenous reaction of the TLRs system on the isolation conditions. It manifested in the diverse dynamics in the absolute and relative content of TLRs^+^ monocytes, as well as their expression of surface and intracellular TLRs. This observation was true for both peripheral blood monocytes and those stimulated with TLRs ligands. Considering the process from the wave model point of view, one can conclude that some endogenous or exogenous stimulation of the receptors of interest might have taken place. Interestingly, the changes of some parameters characterizing the state of the innate immunity, such as the content of TLR2^+^, TLR3^+^, TLR6^+^, TLR8^+^-monocytes, were observed two days before the confinement. This bears evidence of the psychogenic component involvement in the fluctuations revealed, which is realized through the hypothalamic–pituitary–adrenal (HPA) axis. Its activation leads to alterations in microbiota^31^, which in turn could trigger the changes in innate immunity. Also, stress hormones provide cytokine release in immune cells^32^ and cytokines subsequently lead to HSP secretion^33^. The analysis of the stress markers concentrations, namely cortisol, adrenaline, and noradrenaline, could indirectly prove the involvement of HPA in the observed processes. All the molecules tested demonstrated an increase in the concentration by the baseline 2, however, these changes appeared to be insignificant even for adrenaline. The significant increase of adrenaline concentration was observed on the 7th day of the confinement, while cortisol levels declined at the same timepoint. Noradrenaline concentrations were only significantly decreased by the 7th day of the recovery period. Unfortunately, due to a small number of volunteers tested, we observed a high individual variability of the results, which, to a considerable extent, impedes the analysis of the data obtained.

### Gene expression

**Figure 8:**
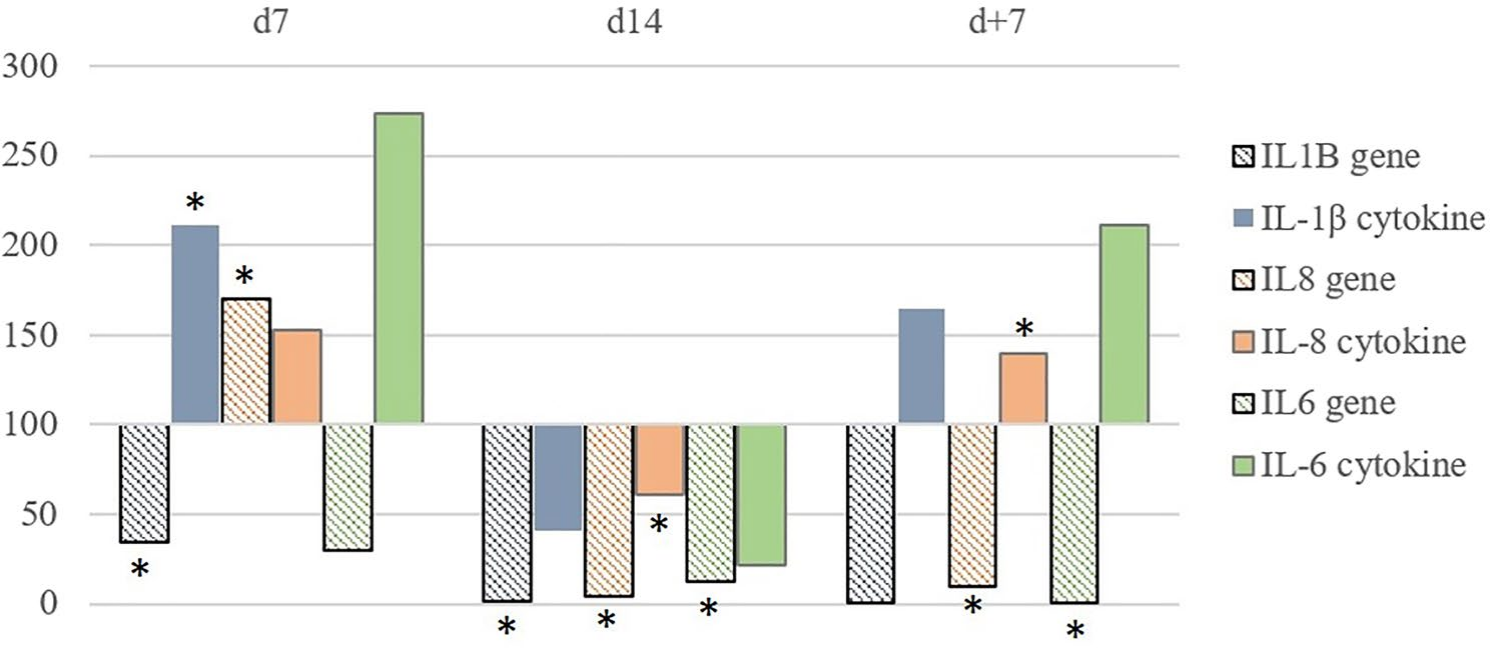
Comparison of cytokine gene and protein expression in test-volunteers during the mission at the indicated timepoints. Gene and protein expression are shown in percent from the values of those in baseline 1 (d-7, taken as 100%). Asterisk (*) indicates *p*-value ≤ 0.05 in Wilcoxon test.

The expression of TLRs genes did not coincide with the expression of the corresponding proteins, while the dynamics of spontaneous cytokine synthesis (IL-6, IL-8, IL-1β) concurred with the expression of the corresponding genes in the timepoints where the changes of the parameters were significant (Fig. 8). Protein synthesis is a multistep process, including the stages of transcription, RNA processing, translation and post-translational modifications of polypeptide chains^34^. As it was shown by Schwanhäusser et al. in 2011^35^, only in 40% of cases the mRNA level determines the variability of protein levels. Nevertheless, the investigation of gene expression as one of the fundamental processes of a live cell functioning is of particular importance to disclose the early immune system reactions to the changing environmental conditions.

### Cytokine production

The dynamics of cytokine concentration in the blood serum were not concurrent with those in cell cultures, which can be explained by the production of cytokines by other immunocompetent cells or non-immune organs and tissues, e.g., muscle or endothelial cells^36,37^.

The most important results of the experiment conducted concern the study of the induced synthesis of cytokines by monocytes in response to the stimulation with the corresponding ligands for TLRs and NLRs. We demonstrated that the conditions of 14-day confinement caused a significant increase of the synthesis of cytokines upon both TLRs and NLRs stimulation preserved by the completion of the experiment. The basal cytokine production by the 24 h-cultured monocytes was also predominantly increased. It may be related to the exchange of microbes in the closed environment, where the immune system is activated. The activation ability of CD14^+^-cells in response to the stimulation with the cocktails of TLRs and NLRs ligands was also investigated during the confinement as a test of the reserve potential of the monocytes. It is noteworthy, that in this experiment the increased production of both pro-inflammatory (GM-CSF, IL-8, etc.) and anti-inflammatory (IL-10) cytokines was observed.

On the whole, we demonstrated that 14-day confinement had no negative effect on the cytokine producing ability of monocytes, moreover, multi-receptor stimulation led to an increased cytokine secretion during the mission and upon its completion compared to baseline. This is unconcordant with the results obtained in the previous experiment of 21-day dry immersion, in which the test volunteers without countermeasures demonstrated the decline in the activation potential of monocytes^38^.

The further accumulation of experimental data in the field of space immunology, their systematization and thorough analysis, as well as the comparison to the data on the state of other physiological systems, especially the neuroendocrine one, will allow to conceptualize the potential mechanisms by which the extreme factors associated with space flight affect human immunity. This will give the necessary ground for the development of novel prospective complex countermeasures and correction strategies for the imbalance and malfunctioning of the immune system.

## Supplementary Information

Supplementary Information.

## Data Availability

The authors confirm that the data supporting the findings of this study are available within the article and its supplementary materials. Additionally, the datasets on gene expression generated and analyzed during the current study have been deposited in NCBI's Gene Expression Omnibus^39^, and are accessible through GEO Series accession number GSE196510 (https://www.ncbi.nlm.nih.gov/geo/query/acc.cgi?acc=GSE196510).
