# Supplementary material for: The impact of short-term confinement on human innate immunity
Source: Sci Rep. 2022 May 19;12:8372. doi: 10.1038/s41598-022-12380-5 (PMC9120181; doi:10.1038/s41598-022-12380-5)
Supplement: Supplementary file 1 — Supplementary Information. [file 41598_2022_12380_MOESM1_ESM.docx]

**The impact of short-term confinement on human innate immunity**

**Ponomarev S.A.^1*^, Sadova A.A.^1,2^, Rykova M.P.^1^, Orlova K.D.^1^, Vlasova D.D.^1^, Shulgina S.M.^1^, Antropova E.N.^1^, Kutko O.V.^1^, Germanov N.S.^1,2^, Galina V.S.^1,2^, Shmarov V.A.^1^**

^1^Laboratory of immune system physiology, SSC RF-IBMP RAS, Moscow, 123007, Russian Federation

^2^Pirigov Russian National Research Medical University (Pirogov Medical University), Moscow, 117997, Russian Federation

*** Correspondence:**

Ponomarev Sergey A.

[dr.grey@bk.ru](mailto:dr.grey@bk.ru)

**Supplementary Table S1. The analysis of monocytes in peripheral blood.**

| **Monocytes** | | | | | | | | | | | | | | | | | | | | |
| --- | --- | --- | --- | --- | --- | --- | --- | --- | --- | --- | --- | --- | --- | --- | --- | --- | --- | --- | --- | --- |
|  |  |  | **baseline -7day** | | | **baseline -2day** | | | **3day** | | | **7day** | | | **14day** | | | **RP +7day** | | |
|  |  |  | **Ме** | **q75** | **q25** | **Ме** | **q75** | **q25** | **Ме** | **q75** | **q25** | **Ме** | **q75** | **q25** | **Ме** | **q75** | **q25** | **Ме** | **q75** | **q25** |
| **CD14** | **%** | | **4,27** | 4,78 | 3,89 | **4,57** | 5,32 | 3,77 | **8,36** | 8,87 | 7,17 | **7,13** | 7,93 | 6,36 | **8,86** | 9,44 | 7,13 | **6,70** | 8,61 | 6,18 |
|  | **abs., 10^9^/л** | | **0,22** | 0,25 | 0,2 | **0,22** | 0,28 | 0,19 | **0,42** | 0,48 | 0,34 | **0,38** | 0,41 | 0,34 | **0,52** | 0,57 | 0,43 | **0,38** | 0,48 | 0,27 |
| **TLR1** | **%** | | **58,28** | 70,62 | 30,68 | **15,26** | 23,29 | 4,97 | **80,12** | 87,22 | 70,86 | **54,13** | 69,28 | 47,96 | **82,36** | 84,20 | 76,27 | **16,00** | 37,51 | 6,03 |
|  | **Expression** | | **1,82** | 2,03 | 1,51 | **1,26** | 1,32 | 1,13 | **2,39** | 2,66 | 2,21 | **2,11** | 2,60 | 1,69 | **2,81** | 3,06 | 2,62 | **1,44** | 1,59 | 1,28 |
|  | **abs., 10^9^/л** | | **0,1** | 0,14 | 0,07 | **0,04** | 0,05 | 0,01 | **0,32** | 0,38 | 0,23 | **0,18** | 0,23 | 0,16 | **0,41** | 0,48 | 0,31 | **0,04** | 0,17 | 0,02 |
| **TLR2** | **%** | | **99,9** | 100,0 | 99,95 | **99,98** | 100,0 | 99,94 | **99,96** | 99,99 | 99,95 | **100,0** | 100,0 | 100,0 | **100,0** | 100,0 | 100,0 | **9,92** | 22,00 | 8,32 |
|  | **Expression** | | **57,24** | 67,35 | 41,21 | **39,50** | 60,33 | 38,09 | **88,01** | 105,24 | 59,41 | **48,37** | 58,84 | 41,55 | **49,86** | 66,21 | 47,26 | **1,48** | 1,78 | 1,33 |
|  | **abs., 10^9^/л** | | **0,22** | 0,25 | 0,20 | **0,22** | 0,28 | 0,19 | **0,42** | 0,48 | 0,34 | **0,38** | 0,41 | 0,34 | **0,52** | 0,57 | 0,43 | **0,03** | 0,11 | 0,03 |
| **TLR3** | **%** | | **0,46** | 0,83 | 0,09 | **0,38** | 0,98 | 0,06 | **1,28** | 2,02 | 0,55 | **1,67** | 2,80 | 0,74 | **0,47** | 0,63 | 0,29 | **0,84** | 1,47 | 0,44 |
|  | **Expression** | | **117,2** | 121,99 | 116,42 | **310,74** | 385,62 | 300,44 | **23,18** | 28,22 | 17,98 | **18,03** | 20,88 | 11,47 | **77,74** | 85,1 | 71,94 | **137,84** | 153,54 | 127,09 |
|  | **abs., 10^9^/л** | | **0,00** | 0,00 | 0,00 | **0,00** | 0,00 | 0,00 | **0,01** | 0,01 | 0,00 | **0,00** | 0,01 | 0,00 | **0,00** | 0,00 | 0,00 | **0,00** | 0,00 | 0,00 |
| **TLR4** | **%** | | **45,34** | 58,75 | 32,31 | **76,25** | 80,01 | 58,59 | **71,18** | 78,86 | 60,09 | **61,12** | 69,17 | 46,7 | **48,02** | 68,15 | 28,83 | **16,42** | 18,83 | 13,6 |
|  | **Expression** | | **2,63** | 3,53 | 2,03 | **4,03** | 4,56 | 3,21 | **3,5** | 4,05 | 3,1 | **3,52** | 6,13 | 2,11 | **3,3** | 7,07 | 2,50 | **1,00** | 1,22 | 0,95 |
|  | **abs., 10^9^/л** | | **0,11** | 0,13 | 0,07 | **0,13** | 0,17 | 0,11 | **0,29** | 0,33 | 0,27 | **0,18** | 0,30 | 0,13 | **0,21** | 0,27 | 0,17 | **0,08** | 0,1 | 0,04 |
| **TLR6** | **%** | | **57,79** | 73,70 | 44,65 | **61,04** | 72,67 | 54,92 | **79,73** | 91,74 | 75,39 | **44,73** | 65,64 | 39,44 | **59,06** | 69,92 | 53,85 | **1,14** | 22,62 | 0,36 |
|  | **Expression** | | **3,59** | 4,38 | 2,35 | **5,04** | 5,44 | 4,66 | **6,38** | 6,73 | 6,27 | **3,65** | 5,26 | 3,39 | **4,90** | 5,33 | 4,79 | **1,23** | 1,29 | 1,17 |
|  | **abs., 10^9^/л** | | **0,11** | 0,16 | 0,11 | **0,14** | 0,18 | 0,11 | **0,34** | 0,38 | 0,3 | **0,16** | 0,19 | 0,14 | **0,31** | 0,40 | 0,23 | **0,00** | 0,07 | 0,00 |
| **TLR8** | **%** | | **35,42** | 47,53 | 14,21 | **0,63** | 0,69 | 0,44 | **8,59** | 13,38 | 5,26 | **12,48** | 35,31 | 7,48 | **85,07** | 88,65 | 82,90 | **99,47** | 99,71 | 99,21 |
|  | **Expression** | | **1,82** | 1,85 | 1,81 | **1,22** | 1,24 | 1,21 | **1,56** | 1,62 | 1,51 | **1,42** | 1,55 | 1,39 | **2,53** | 2,74 | 2,32 | **7,72** | 8,07 | 7,29 |
|  | **abs., 10^9^/л** | | **0,06** | 0,09 | 0,03 | **0,00** | 0,00 | 0,00 | **0,03** | 0,04 | 0,02 | **0,02** | 0,15 | 0,02 | **0,16** | 0,19 | 0,14 | **0,16** | 0,22 | 0,14 |
| **TLR9** | **%** | | **3,63** | 4,19 | 2,56 | **0,70** | 1,16 | 0,50 | **2,04** | 5,20 | 1,29 | **5,34** | 6,73 | 4,95 | **31,41** | 36,18 | 27,93 | **36,93** | 37,65 | 35,09 |
|  | **Expression** | | **1,35** | 1,44 | 1,25 | **1,23** | 1,24 | 1,21 | **1,44** | 1,53 | 1,30 | **1,42** | 1,45 | 1,23 | **1,80** | 1,89 | 1,73 | **2,21** | 2,33 | 1,99 |
|  | **abs., 10^9^/л** | | **0,01** | 0,01 | 0,01 | **0,00** | 0,00 | 0,00 | **0,01** | 0,02 | 0,01 | **0,01** | 0,03 | 0,01 | **0,07** | 0,08 | 0,05 | **0,06** | 0,10 | 0,05 |

|  | Significant difference with baseline 1 | | p<0,05 |  |
| --- | --- | --- | --- | --- |
|  | |  |  |  |
|  | |  |  |  |

**Supplementary Table S2. The analysis of TLRs expression in cultured monocytes stimulated with TLRs ligands**

| **Cellular cultures** | | | | | | | | | | | | | | | | | |
| --- | --- | --- | --- | --- | --- | --- | --- | --- | --- | --- | --- | --- | --- | --- | --- | --- | --- |
|  |  |  | **baseline -7day** | | | **3day** | | | **7day** | | | **14day** | | | **RP +7day** | | |
|  |  |  | **Ме** | **q75** | **q25** | **Ме** | **q75** | **q25** | **Ме** | **q75** | **q25** | **Ме** | **q75** | **q25** | **Ме** | **q75** | **q25** |
| **TLR1** | **%** | **Cont** | **40,48** | 49,94 | 17,39 | **28,73** | 37,96 | 27,60 | **30,68** | 38,11 | 17,70 | **64,29** | 76,54 | 58,39 | **57,77** | 79,50 | 37,19 |
|  |  | **Exp** | **51,82** | 65,08 | 45,24 | **80,08** | 94,83 | 70,06 | **46,52** | 72,59 | 24,38 | **66,84** | 70,42 | 54,56 | **70,66** | 76,93 | 67,10 |
|  | **Expression** | **Cont** | **2,08** | 2,79 | 1,21 | **1,66** | 2,67 | 1,46 | **1,92** | 2,15 | 1,76 | **3,05** | 3,52 | 2,62 | **3,87** | 3,93 | 3,77 |
|  |  | **Exp** | **2,91** | 3,61 | 2,39 | **3,52** | 3,85 | 3,02 | **2,35** | 3,07 | 1,75 | **2,95** | 3,02 | 2,65 | **3,67** | 4,07 | 3,51 |
| **TLR2** | **%** | **Cont** | **95,91** | 96,59 | 90,01 | **87,54** | 98,41 | 62,50 | **77,13** | 91,94 | 55,91 | **97,31** | 98,85 | 94,02 | **31,14** | 43,92 | 17,78 |
|  |  | **Exp** | **98,07** | 99,06 | 93,30 | **85,84** | 97,14 | 81,82 | **77,13** | 97,33 | 54,56 | **97,45** | 98,96 | 96,20 | **36,10** | 39,93 | 18,56 |
|  | **Expression** | **Cont** | **6,61** | 7,78 | 5,96 | **8,91** | 17,40 | 3,67 | **6,34** | 8,00 | 5,16 | **9,62** | 10,26 | 8,79 | **2,92** | 3,30 | 2,26 |
|  |  | **Exp** | **10,15** | 20,59 | 7,54 | **7,01** | 19,12 | 5,91 | **8,30** | 9,74 | 5,01 | **12,26** | 14,19 | 10,55 | **3,07** | 3,55 | 2,46 |
| **TLR3** | **%** | **Cont** | **24,37** | 35,99 | 14,85 | **24,65** | 38,78 | 3,35 | **46,86** | 57,49 | 40,19 | **77,83** | 82,80 | 43,02 | **97,87** | 98,42 | 96,83 |
|  |  | **Exp** | **29,06** | 76,58 | 20,78 | **58,77** | 65,21 | 14,15 | **45,95** | 62,23 | 38,69 | **68,00** | 74,82 | 60,65 | **96,55** | 97,82 | 93,70 |
|  | **Expression** | **Cont** | **1,41** | 1,50 | 1,39 | **1,92** | 2,18 | 1,23 | **1,76** | 2,02 | 1,69 | **2,16** | 2,20 | 2,07 | **6,19** | 6,40 | 5,88 |
|  |  | **Exp** | **1,56** | 1,58 | 1,32 | **1,68** | 1,95 | 1,67 | **1,76** | 1,91 | 1,64 | **2,18** | 2,38 | 2,10 | **5,37** | 6,03 | 5,13 |
| **TLR4** | **%** | **Cont** | **94,22** | 98,33 | 89,62 | **92,83** | 97,17 | 88,39 | **87,02** | 92,49 | 61,93 | **99,54** | 99,68 | 99,40 | **70,56** | 86,68 | 62,58 |
|  |  | **Exp** | **99,20** | 99,89 | 95,06 | **84,89** | 99,16 | 50,00 | **86,28** | 92,83 | 60,15 | **99,42** | 99,54 | 98,06 | **60,01** | 63,51 | 31,69 |
|  | **Expression** | **Cont** | **5,86** | 20,91 | 4,96 | **19,36** | 49,31 | 13,91 | **7,13** | 8,00 | 6,52 | **22,77** | 25,49 | 21,00 | **5,97** | 7,77 | 4,58 |
|  |  | **Exp** | **11,12** | 26,24 | 6,65 | **17,42** | 45,18 | 9,67 | **8,28** | 10,98 | 6,46 | **47,82** | 53,91 | 40,89 | **5,22** | 6,79 | 4,83 |
| **TLR6** | **%** | **Cont** | **5,47** | 9,18 | 4,18 | **49,47** | 72,41 | 22,55 | **4,04** | 5,33 | 2,78 | **32,49** | 43,23 | 24,97 | **2,49** | 5,72 | 0,88 |
|  |  | **Exp** | **34,57** | 54,57 | 18,19 | **29,29** | 90,13 | 21,79 | **6,90** | 7,64 | 4,23 | **20,22** | 54,17 | 8,89 | **2,32** | 2,56 | 1,64 |
|  | **Expression** | **Cont** | **1,12** | 1,15 | 1,04 | **1,71** | 7,68 | 1,36 | **1,10** | 1,26 | 1,08 | **2,06** | 2,61 | 1,77 | **1,47** | 1,55 | 1,46 |
|  |  | **Exp** | **2,00** | 2,43 | 1,47 | **1,91** | 11,44 | 1,68 | **1,20** | 1,31 | 1,12 | **1,72** | 3,02 | 1,40 | **1,57** | 1,46 | 1,42 |
| **TLR8** | **%** | **Cont** | **0,39** | 0,46 | 0,24 | **43,50** | 46,06 | 22,56 | **26,91** | 33,51 | 23,47 | **48,77** | 54,11 | 25,17 | **21,84** | 24,50 | 16,20 |
|  |  | **Exp** | **1,40** | 27,40 | 1,07 | **71,81** | 76,05 | 18,38 | **24,69** | 53,78 | 17,66 | **36,37** | 55,13 | 29,80 | **20,97** | 25,23 | 19,38 |
|  | **Expression** | **Cont** | **1,00** | 1,05 | 0,95 | **2,51** | 2,51 | 2,14 | **1,89** | 2,41 | 1,74 | **2,21** | 2,56 | 2,19 | **12,76** | 16,54 | 10,76 |
|  |  | **Exp** | **1,00** | 1,05 | 0,94 | **2,77** | 3,78 | 2,29 | **1,68** | 2,41 | 1,65 | **2,18** | 2,29 | 2,11 | **17,46** | 19,30 | 12,85 |
| **TLR9** | **%** | **Cont** | **3,25** | 8,25 | 2,56 | **5,14** | 5,97 | 4,56 | **14,77** | 16,22 | 11,73 | **29,30** | 51,90 | 15,68 | **91,02** | 91,84 | 89,96 |
|  |  | **Exp** | **8,60** | 46,77 | 2,46 | **30,27** | 32,41 | 12,57 | **15,83** | 49,51 | 15,19 | **29,42** | 40,82 | 17,27 | **78,69** | 82,78 | 76,88 |
|  | **Expression** | **Cont** | **1,20** | 1,35 | 1,17 | **1,50** | 1,82 | 1,38 | **1,27** | 1,46 | 1,27 | **1,59** | 1,63 | 1,55 | **4,84** | 5,85 | 4,10 |
|  |  | **Exp** | **1,25** | 1,43 | 1,07 | **1,61** | 3,17 | 1,32 | **1,37** | 1,63 | 1,33 | **1,64** | 1,67 | 1,62 | **3,37** | 5,48 | 2,71 |

|  | Significant difference with baseline 1 | p<0,05 |
| --- | --- | --- |

**Supplementary Table S3. Induced cytokine synthesis in cultured monocytes stimulated with corresponding ligands for surface TLRs.**

|  | | **GM-CSF** | **IFN-α2** | **IFN-y** | **IL-10** | **IL-12P40** | **IL-12P70** | **IL-1β** | **IL-6** | **IL-8** | **TNF-α** |
| --- | --- | --- | --- | --- | --- | --- | --- | --- | --- | --- | --- |
| **Control** | baseline 1 | 7,87 | 31,11 | 2,64 | 10,66 | 13,36 | 2,70 | 36,30 | 323,93 | 6495,50 | 277,78 |
|  | 3d | 9,57 | 11,62 | 2,92 | 2,01 | 8,14 | 1,15 | **24,94** | 76,63 | 1021,00 | 107,21 |
|  | 7d | **15,12** | 34,84 | 3,78 | **43,22** | 16,04 | 4,05 | 76,69 | 886,78 | **9886,00** | 280,27 |
|  | 14d | 8,90 | **18,37** | 1,85 | 4,79 | 9,87 | 1,37 | **14,99** | **69,21** | 3958,84 | **59,64** |
|  | RP +7d | 24,00 | 32,26 | 3,51 | 26,62 | 15,25 | 3,10 | 59,62 | 683,03 | 9088,50 | 170,90 |
| **TLR1** | baseline 1 | 22,76 | 35,58 | 3,63 | 71,14 | 20,68 | 4,19 | 75,41 | 2169,50 | 13175,20 | 1226,00 |
|  | 3d | 10,55 | 21,00 | 2,71 | 2,88 | 9,52 | 2,38 | 22,74 | 105,52 | 1151,00 | 164,75 |
|  | 7d | **51,63** | **48,12** | 5,79 | **376,99** | 34,87 | 5,82 | **217,87** | 4308,00 | 39460,50 | 1849,50 |
|  | 14d | **40,71** | **41,31** | 4,50 | 151,13 | 23,85 | 4,20 | 85,90 | 3908,00 | 22426,00 | 1020,00 |
|  | RP +7d | **82,58** | **50,13** | **5,69** | **380,04** | **27,68** | 5,00 | **302,58** | 8966,00 | 63626,50 | 1645,50 |
| **TLR2** | baseline 1 | 14,44 | 31,08 | 3,55 | 48,08 | 13,73 | 3,07 | 44,86 | 522,65 | 7736,52 | 512,06 |
|  | 3d | 5,25 | 16,19 | 2,50 | 3,10 | 8,14 | 2,38 | 26,72 | 124,20 | 1806,00 | 176,17 |
|  | 7d | **63,94** | **51,82** | **6,05** | **508,26** | **38,07** | **6,04** | **233,61** | **3567,50** | **18055,00** | **2560,00** |
|  | 14d | **41,91** | 44,76 | 4,40 | **203,51** | **26,88** | 4,01 | **109,00** | **1143,00** | **18316,00** | 580,34 |
|  | RP +7d | **125,88** | 59,63 | 5,65 | **742,83** | **46,86** | 6,57 | **505,67** | **8252,00** | **55917,48** | 1997,50 |
| **TLR4** | baseline 1 | 66,55 | 38,21 | 4,21 | 131,98 | 27,05 | 10,19 | 263,59 | 4895,00 | 19993,76 | 3117,00 |
|  | 3d | 7,63 | 11,23 | 2,04 | 2,27 | 6,81 | 1,10 | 32,45 | 191,63 | 2318,00 | 514,50 |
|  | 7d | **123,93** | 54,59 | **7,87** | **1458,00** | **49,61** | 17,04 | **2174,50** | **9837,00** | 17316,50 | 6941,00 |
|  | 14d | 180,90 | 55,88 | **6,21** | **1490,00** | **42,73** | 7,39 | **5245,00** | **9065,00** | 74822,19 | **4727,00** |
|  | RP +7d | **310,45** | 65,00 | **7,30** | **2779,00** | **58,59** | 13,35 | **9767,00** | **14628,58** | **149004,43** | **7520,50** |
| **TLR6** | baseline 1 | 20,24 | 34,26 | 3,51 | 33,83 | 18,19 | 4,01 | 110,52 | 1574,00 | 14214,50 | 750,68 |
|  | 3d | 5,25 | 11,23 | 2,04 | 1,32 | 5,70 | 0,52 | 18,86 | 114,55 | 1511,00 | 182,07 |
|  | 7d | **73,44** | **44,60** | **6,65** | **430,61** | 27,84 | 5,82 | **501,95** | **7802,00** | 16951,00 | **2334,00** |
|  | 14d | 34,64 | 42,65 | 4,07 | 97,72 | 28,87 | 3,81 | 94,46 | 1815,50 | 16753,50 | **388,12** |
|  | RP +7d | **81,98** | **47,32** | **6,14** | 280,22 | 32,66 | 5,40 | **222,09** | **7526,00** | 39059,00 | 864,25 |

|  | Significant difference with baseline 1 | p<0,05 |
| --- | --- | --- |

**Supplementary Table S4. Induced cytokine synthesis in cultured monocytes stimulated with corresponding ligands for intracellular TLRs.**

|  | | **GM-CSF** | **IFN-α2** | **IFN-y** | **IL-10** | **IL-12P40** | **IL-12P70** | **IL-1β** | **IL-6** | **IL-8** | **TNF-α** |
| --- | --- | --- | --- | --- | --- | --- | --- | --- | --- | --- | --- |
| **Control** | baseline 1 | 7,87 | 31,11 | 2,64 | 10,66 | 13,36 | 2,70 | 36,30 | 323,93 | 6495,50 | 277,78 |
|  | 3d | 9,57 | 11,62 | 2,92 | 2,01 | 8,14 | 1,15 | **24,94** | 76,63 | 1021,00 | 107,21 |
|  | 7d | **15,12** | 34,84 | 3,78 | **43,22** | 16,04 | 4,05 | 76,69 | 886,78 | **9886,00** | 280,27 |
|  | 14d | 8,90 | **18,37** | 1,85 | 4,79 | 9,87 | 1,37 | **14,99** | **69,21** | 3958,84 | **59,64** |
|  | RP +7d | 24,00 | 32,26 | 3,51 | 26,62 | 15,25 | 3,10 | 59,62 | 683,03 | 9088,50 | 170,90 |
| **TLR3** | baseline 1 | 11,06 | 28,05 | 2,96 | 2,96 | 19,90 | 2,96 | 48,35 | 416,18 | 7015,00 | 363,17 |
|  | 3d | 7,74 | 18,62 | 3,06 | 3,06 | 12,82 | 1,63 | 30,74 | 105,93 | 1346,00 | 148,60 |
|  | 7d | 20,47 | 34,84 | **5,93** | 5,93 | 35,94 | **6,33** | 75,49 | 864,85 | 7632,50 | 402,28 |
|  | 14d | 13,98 | 23,65 | 2,48 | 2,48 | 14,15 | 1,90 | **24,33** | 233,88 | 6144,00 | **115,45** |
|  | RP +7d | 29,21 | 39,24 | **6,00** | 6,00 | **33,50** | 3,83 | 66,48 | 595,87 | 10819,00 | 172,64 |
| **TLR8** | baseline 1 | 9,57 | 29,51 | 3,07 | 18,68 | 13,57 | 4,61 | 92,93 | 484,31 | 7352,50 | 475,61 |
|  | 3d | 5,25 | 11,62 | 2,04 | 1,32 | 5,70 | 1,15 | 24,41 | 110,23 | 1584,00 | 147,04 |
|  | 7d | **44,66** | **53,66** | **5,17** | **331,74** | **35,27** | **20,40** | **750,00** | **4361,00** | 20190,00 | **1894,50** |
|  | 14d | **25,03** | 38,56 | 3,80 | 46,35 | 20,93 | 6,04 | 136,79 | 554,26 | 10512,50 | 249,41 |
|  | RP +7d | **31,30** | 39,93 | **5,26** | **75,48** | 28,09 | 5,78 | 189,92 | 1374,50 | 22157,50 | 299,84 |
| **TLR9** | baseline 1 | 11,16 | 31,11 | 2,92 | 24,82 | 13,57 | 2,70 | 52,90 | 659,37 | 8706,00 | 434,46 |
|  | 3d | 12,02 | **11,23** | 2,04 | 2,27 | 6,16 | 1,37 | 32,19 | 322,70 | **4548,00** | 409,56 |
|  | 7d | 42,34 | 39,09 | **5,17** | **219,33** | 22,27 | 6,24 | 282,45 | 3056,00 | 25523,50 | **1022,15** |
|  | 14d | 13,97 | 32,55 | 3,21 | 8,23 | 15,48 | 2,37 | **22,43** | **154,40** | 6797,61 | 91,87 |
|  | RP +7d | 36,12 | 39,24 | 4,70 | 33,16 | **23,05** | 3,22 | 84,11 | 1053,56 | 11366,00 | 186,26 |
| **TLR mix** | baseline 1 | 93,06 | 51,43 | 5,17 | 521,72 | 37,16 | 16,13 | 348,43 | 8445,00 | 19535,50 | 5630,50 |
|  | 7d | 140,47 | 58,31 | **10,82** | **2624,00** | 54,35 | 24,53 | 3119,00 | 11810,00 | 125864,00 | 8597,50 |
|  | 14d | 288,65 | 57,12 | 7,27 | 2156,00 | 54,78 | 9,17 | 6090,00 | 9657,50 | 27338,50 | 5282,50 |
|  | RP +7d | **273,67** | 63,25 | 7,78 | **2928,00** | 50,83 | 10,84 | 8372,50 | 12430,00 | 131327,00 | 6925,50 |

|  | Significant difference with baseline 1 | p<0,05 |
| --- | --- | --- |
